# Maternal Melatonin Therapy Attenuated Maternal High-Fructose Combined with Post-Weaning High-Salt Diets-Induced Hypertension in Adult Male Rat Offspring

**DOI:** 10.3390/molecules23040886

**Published:** 2018-04-11

**Authors:** You-Lin Tain, Steve Leu, Wei-Chia Lee, Kay L. H. Wu, Julie Y. H. Chan

**Affiliations:** 1 Department of Pediatrics, Kaohsiung Chang Gung Memorial Hospital and Chang Gung University College of Medicine, Kaohsiung 833, Taiwan; tainyl@hotmail.com; 2 Institute for Translational Research in Biomedicine, Kaohsiung Chang Gung Memorial Hospital and Chang Gung University College of Medicine, Kaohsiung 833, Taiwan; leu@mail.cgu.edu.tw (S.L.); wlh0701@yahoo.com (K.L.H.W.); 3 Department of Urology, Kaohsiung Chang Gung Memorial Hospital and Chang Gung University College of Medicine, Kaohsiung 833, Taiwan; dinor666@ms32.hinet.net

**Keywords:** asymmetric dimethylarginine, developmental origins of health and disease (DOHaD), fructose, hypertension, melatonin, nutrient sensing signal, salt

## Abstract

Consumption of food high in fructose and salt is associated with the epidemic of hypertension. Hypertension can originate from early life. Melatonin, a pleiotropic hormone, regulates blood pressure. We examined whether maternal melatonin therapy can prevent maternal high-fructose combined with post-weaning high-salt diet-induced programmed hypertension in adult offspring. Pregnant Sprague-Dawley rats received either a normal diet (ND) or a 60% fructose diet (HF) during pregnancy and the lactation period. Male offspring were on either the ND or a high-salt diet (HS, 1% NaCl) from weaning to 12 weeks of age and were assigned to five groups (n = 8/group): ND/ND, HF/ND, ND/HS, HF/HS, and HF/HS+melatonin. Melatonin (0.01% in drinking water) was administered during pregnancy and lactation. We observed that maternal HF combined with post-weaning HS diets induced hypertension in male adult offspring, which was attenuated by maternal melatonin therapy. The beneficial effects of maternal melatonin therapy on HF/HS-induced hypertension related to regulating several nutrient-sensing signals, including *Sirt1*, *Sirt4*, *Prkaa2*, *Prkab2*, *Pparg*, and *Ppargc1a*. Additionally, melatonin increased protein levels of mammalian targets of rapamycin (mTOR), decreased plasma asymmetric dimethylarginine (ADMA) and symmetric dimethylarginine levels, and increased the l-arginine-to-ADMA ratio. The reprogramming effects by which maternal melatonin therapy protects against hypertension of developmental origin awaits further elucidation.

## 1. Introduction

Melatonin, a pineal indole hormone, has pleiotropic bioactivities, including regulating circadian rhythm, redox homeostasis, epigenetic regulation, glucose metabolism, anti-inflammation and anti-aging actions, and fetal development [1,2,3,4,5]. Additionally, experimental and human studies have indicated that melatonin can regulate blood pressure (BP) [6,7].

The origins of susceptibility for hypertension in adults can be traced back to early life, formally named as the “developmental origins of health and disease” (DOHaD) [8]. This concept has also led to a shift in therapeutic approaches from adulthood to early life, before hypertension is evident, by so-called reprogramming [9]. A growing body of experimental studies supports the notion that melatonin may act as a key component of reprogramming strategies to prevent a variety of diseases, including hypertension [10].

An imbalanced diet is a major environmental insult in gene–environment interactions underlying hypertension of developmental origin. Diets laden with refined sugars and salt have been implicated in the pathogenesis of hypertension, and there is a synergistic effect between sugars and salt on BP elevation [11]. Fructose consumption, mainly from dietary refined sugars, has risen steeply in the last half-century [12]. Consumption of high-fructose (HF) diets by rodent mothers causes programmed hypertension in their adult offspring [13]. Our previous report showed that adult offspring of mothers exposed to 60% HF diet during pregnancy and lactation developed hypertension, which can be exacerbated by a post-weaning high-salt diet [14]. However, whether maternal melatonin therapy is able to prevent programmed hypertension in adult offspring induced by maternal HF combined with post-weaning high-salt (HS) diets remains unknown.

Nutrient-sensing signals play a key role in fetal development [15]. Maternal and postnatal nutritional insults may disturb these sensing signals during the critical developmental phase that leads to hypertension of developmental origin [16]. Our previous study suggested that nutrient-sensing signals are crucial for the response of different organs of offspring to maternal HF consumption for programming differential phenotypes of metabolic syndrome, including hypertension [13]. Additionally, BP was controlled by nitric oxide (NO) systems. Asymmetric dimethylarginine (ADMA), an endogenous NO synthase inhibitor, can regulate the NO-reactive oxygen species (ROS) balance, and is involved in the development of hypertension [17]. Reprogramming interventions aimed at NO-ROS balance has been reported to be protective in developmentally programmed hypertension [9,18].

BP is regulated by a complex process that is governed mainly by the kidney. The developing kidney is vulnerable to adverse early-life environments, which may produce long-term effects on the structure and/or function of the kidney by so-called renal programming. Despite progress made in last five decades on identifying melatonin and its multiple biological functions, few studies have targeted its potential for a reprogramming strategy against hypertension of developmental origin. Therefore, we examined whether maternal melatonin therapy can reprogram disturbed nutrient-sensing signals and ADMA/NO pathway, by which it protects against developmentally programmed hypertension induced by combined maternal HF and post-weaning HS diets, with a focus on the kidney. 

## 2. Results

**Figure 1:**
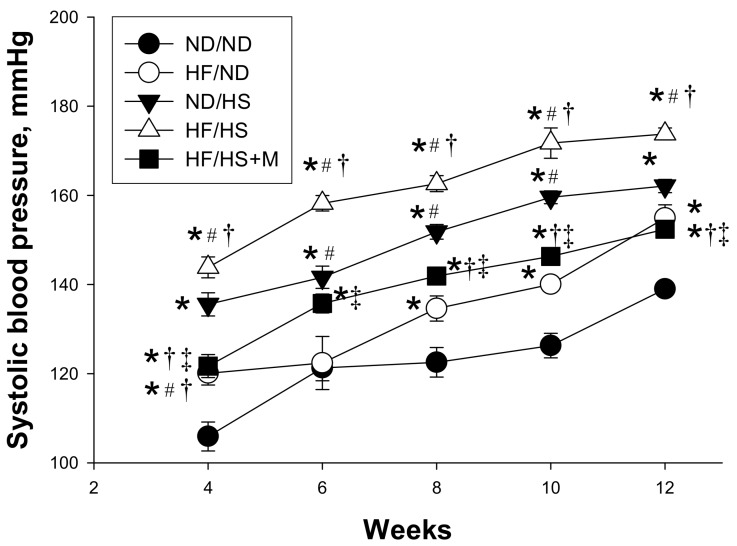
Effect of maternal high-fructose (HF) diet, post-weaning high-salt (HS) diet, and melatonin (M) on systolic blood pressure in 12-week-old male offspring. * *p* < 0.05 vs. ND/ND, ^#^ *p* < 0.05 vs. HF/ND, ^†^ *p* < 0.05 vs. ND/HS, ^‡^ *p* < 0.05 vs. HF/HS.

There was no difference in the mortality rate of the male pups among the five groups (Table 1). The ND/HS and HF/HS groups had a greater body weight (BW) compared with the other three groups. Similarly, the kidney weights were higher in the ND/HS and HF/HS groups compared to ND/ND, HF/ND, and HF/HS+M groups. However, the kidney weight-to-body weight ratios were no different among the five groups. The systolic BP of HF/ND group was significantly higher than that in the ND/ND group at 12 weeks of age. Post-weaning HS diet caused a marked increase in systolic BP (SBP, ∼20 mm Hg). This increase in SBP was reduced (∼20 mm Hg) by maternal melatonin therapy. Similarly, the diastolic BP and mean arterial pressure were highest in the HF/HS group compared to the other four groups, which melatonin prevented. As shown in Figure 1, SBP significantly increased in ND/HS group compared with that in ND/ND group from 4 to 12 weeks of age, and was the highest in the HF/HS group. These data indicated that post-weaning HS intake aggravated maternal HF-induced programmed hypertension, which was alleviated by maternal melatonin therapy.

**Figure 2:**
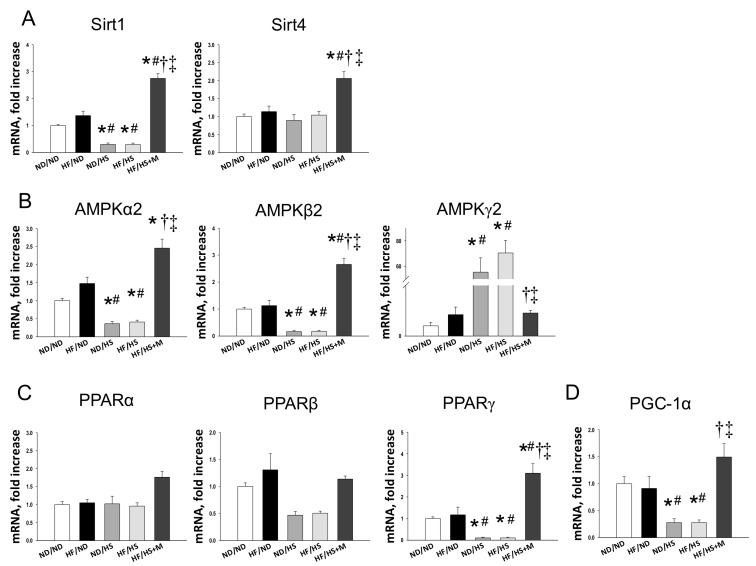
Effect of maternal and post-weaning high fructose (HF) intake, post-weaning high salt (HS) intake, and melatonin (M) on mRNA expression of (**A**) silent information regulator transcript 1 (SIRT1) and 4 (SIRT4); (**B**) AMP-activated protein kinase (AMPK) α-, β- and γ-subunits; and (**C**) Peroxisome proliferator-activated receptor (PPAR) α-, β- and γ-isoforms, and (**D**) PPARγ coactivator-1α (PGC-1α) in male offspring kidneys at 12 weeks of age. * *p* < 0.05 vs. ND/ND, # *p* < 0.05 vs. HF/ND, † *p* < 0.05 vs. ND/HS, ‡ *p* < 0.05 vs. HF/HS.

**Figure 3:**
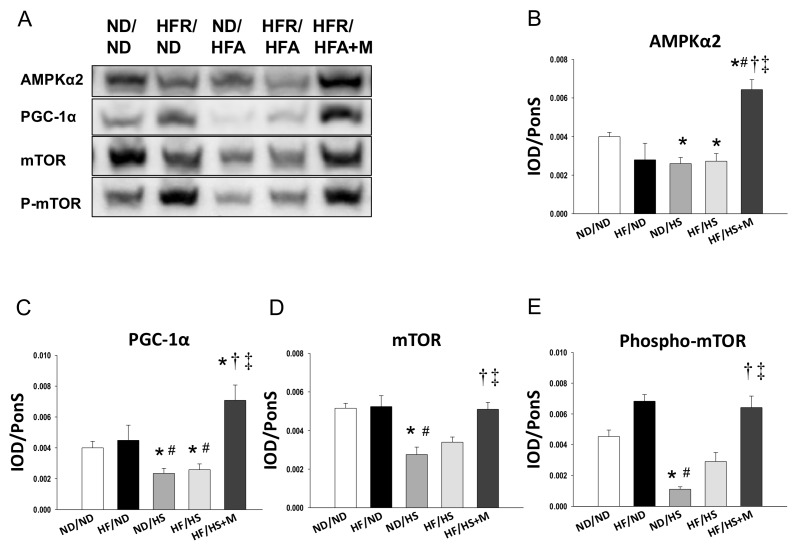
(**A**) Representative Western blots and relative abundance of (**B**) AMPKα2 (63 kDa); (**C**) PGC-1α (90 kDa); (**D**) mammalian target of rapamycin (mTOR, 289kDa) and (**E**) phosphorylated mTOR in male offspring kidneys at 12 weeks of age. * *p* < 0.05 vs. ND/ND, ^#^ *p* < 0.05 vs. HF/ND, ^†^ *p* < 0.05 vs. ND/HS, ^‡^ *p* < 0.05 vs. HF/HS.

We first analyzed genes in the nutrient-sensing pathway, including silent information regulator transcript (SIRT), cyclic adenosine monophosphate (AMP)-activated protein kinase (AMPK), peroxisome proliferator-activated receptors (PPARs), PPARγ coactivator-1α (PGC-1α), and the mammalian target of rapamycin (mTOR) [17]. As shown in Figure 2, renal mRNA expression of *Sirt1*, *Prkaa2* (encoding for AMPKα2), *Prkab2* (encoding for AMPKβ2), *Pparg* (encoding for PPARγ), and *Ppargc1a* (encoding for PGC-1α) in ND/HS and HF/HS rats were lower, while *Prkag2* (encoding for AMPKγ2) were higher than those in ND/ND rats. Maternal melatonin therapy significantly increased mRNA expression of *Sirt1*, *Sirt4*, *Prkaa2*, *Prkab2*, *Pparg*, and *Ppargc1a* in offspring kidneys. Additionally, the increase of mRNA level of *Prkag2* was in HF/HS group was restored by melatonin. Consistent with the change in mRNA level, renal protein levels of AMPKα2 (Figure 3B) and PGC-1α (Figure 3C) were decreased in ND/HS and HF/HS group. These changes were restored by maternal melatonin therapy.

Since autophagy, an evolutionarily conserved catabolic process in maintaining cellular nutrient homeostasis, is regulated by the above-mentioned nutrient-sensing pathways and mTOR [19,20], we further evaluated mTOR signaling. As shown in Figure 3, high-salt diet significantly decreased renal protein levels of mTOR (Figure 3D) and phosphorylated mTOR (Figure 3E), which was restored by maternal melatonin therapy. Similarly, we found mRNA expression of the nuclear factor erythroid-derived 2-related factor 2 (*Nrf2*), a key nutrient sensitive transcription factor in the regulation of oxidative stress response [21], was lower in ND/HS (0.37 ± 0.05-fold change, *p* < 0.05) and HF/HS (0.44 ± 0.04 fold change, *p* < 0.05) group compared to ND/ND group. While this reduction was preserved by melatonin treatment (2.31 ± 0.42 fold change). Taken together, our findings suggest that maternal melatonin therapy reprograms HF/HS-induced renal programming, and that programmed hypertension is associated with mediation of several nutrient-sensing signals and activating mTOR signaling and *Nrf2*.

We further examined NO pathway as the ADMA-NO imbalance contributes to the development of programmed hypertension [17]. As shown in Table 2, post-weaning high-salt diet caused increases of plasma l-citrulline levels in ND/HS and HF/HS group, whereas this increase was prevented by maternal melatonin therapy. Additionally, plasma l-arginine levels were lower in HF/ND and HF/HS+M group compared to those in the ND/ND group. We observed that post-weaning high-salt diet caused plasma ADMA and SDMA levels to exhibit nearly 2- and 4-fold increases compared to the ND/ND group, respectively. These increases were offset by maternal melatonin therapy. Moreover, the l-arginine-to-ADMA ratio was reduced by post-weaning high salt intake in ND/HS and HF/HS rats vs. ND/ND rats. The reduction of the l-arginine-to-ADMA ratio was restored by melatonin. 

## 3. Discussion

Our study provides new insight into how maternal melatonin therapy acts as part of a reprogramming strategy to prevent adult male offspring against programmed hypertension induced by maternal high fructose intake plus post-weaning high-salt diets. The key findings of our study can be summarized as follows: (1) maternal high-fructose diet combined with post-weaning high-salt diet caused hypertension in male adult offspring and maternal melatonin therapy was found to attenuate the development of hypertension in these offspring; (2) maternal melatonin therapy protects against HF/HS-induced hypertension, and is related to increased mRNA expression of *Sirt1*, *Sirt4*, *Prkaa2*, *Prkab2*, *Pparg*, and *Ppargc1a* in offspring kidneys; (3) high-salt diet reduced protein levels of AMPKα2, PGC-1α, mTOR and phosphorylated mTOR, whereas these decreases were restored by maternal melatonin therapy; and (4) maternal melatonin therapy restored the HF/HS-induced increases of plasma l-citrulline, ADMA, and SDMA levels, and the decreases of l-arginine-to-ADMA ratios induced by HF/HS intake.

In support of the notion that pre- and post-natal insults could synergistically contribute to renal programming and programmed hypertension [14,18], our results demonstrated that maternal HF intake, post-weaning HS consumption both synergistically induced programmed hypertension in adult male offspring. Of note, the increases of BPs were mitigated by maternal melatonin therapy. Regardless of the fact that melatonin has been reported to prevent the increase in BPs in adults [6,7], few studies are available regarding dams exposed to melatonin protecting hypertension of developmental origin in their adult offspring [4]. As we show in the present study, maternal melatonin therapy protects against programmed hypertension, and to our knowledge, this is the first report of adult offspring exposed to maternal high-fructose diet combined with post-weaning high-salt diets. Additionally, in agreement with previous studies, our results show mother rats fed with 60% high-fructose diet appear to have a negligible effect on the body weight of their offspring [14,22,23]. It is noteworthy that adverse effects of fructose feeding depend on the amount and duration of fructose consumption [24]. Although an obesogenic effect of maternal HF intake on offspring was observed in some animal studies [25], not all studies in rodents have demonstrated deleterious effects of excess fructose consumption [23].

So far, some particular mechanisms contributing to the reprogramming effects of melatonin have been suggested, such as reduction of oxidative stress, epigenetic regulation, and alterations of the renin-angiotensin system [4,18]. In this work, we observed, for the first time, that that maternal melatonin therapy prevents hypertension associated with increased expression of *Sirt1*, *Sirt4*, *Prkaa2*, *Prkab2*, *Pparg*, and *Ppargc1a* in adult offspring kidneys. This is consistent with our recent report showing that resveratrol, a known AMPK activator, protects against maternal combined with post-weaning high-fat diets-induced hypertension via increasing SIRT1 and AMPKα2 [26]. Additionally, our findings are in agreement with emerging evidence indicating an increase in expression and/or activity of SIRT1 after melatonin treatment [27]. We have previously demonstrated that maternal nutritional insults mediate nutrient-sensing mechanisms to regulate PPAR target genes, contributing to programmed hypertension [28]. As clinical and experimental evidence suggests that administering PPARγ agonists are protective against hypertension [28,29], our data implied that maternal melatonin therapy inducing *Pparg* to regulate its target genes might be a beneficial mechanism, at least in part, by which melatonin reprograms hypertension of developmental origin. Furthermore, we observed that maternal melatonin treatment activates mTOR signals in offspring kidneys. Melatonin has been shown to activate mTOR signaling and prevent ischemic brain injury [30]. Given that mTOR regulates autophagy and that inhibition of autophagy links to hypotensive effects [31,32], our findings suggest that maternal melatonin therapy protects against HF/HS-induced programmed hypertension may be due to inhibition of autophagy via activation of mTOR signals, although this remains speculative.

In the current study, another protective effect of maternal melatonin therapy on hypertension is mediating NO system. We found that HF/HS rats had a higher plasma l-citrulline level compared to those in ND/ND rats and this increase was preserved by melatonin. This is in line with previous observations showing that NO deficiency due to impaired l-citrulline conversion to l-arginine contributes to hypertension [33,34]. Our recent studies indicated that maternal melatonin therapy protects against hypertension is related to reduction of plasma levels of ADMA and SDMA and increases of l-arginine-to-ADMA ratio. Because, individually, ADMA and SDMA are inhibitors of NO synthase and the l-arginine-to-ADMA ratios represent NO bioavailability [35], our data are in agreement with previous studies showing that early treatment with melatonin could restore the ADMA–NO pathway prior to hypertension in favor of NO, to prevent the development of hypertension in spontaneously hypertensive rats [17,36]. Additionally, our current study shows that maternal melatonin therapy significantly increased *Nrf2* expression. As we have recently demonstrated in other work [37], the NRF2 activator dimethyl fumarate reversed combined prenatal dexamethasone exposure and post-weaning high-fat diet-induced increases of ADMA and SDMA concentrations and a decrease in the l-arginine-to-ADMA ratio. Our data suggest that maternal melatonin therapy might activate *Nrf2* signaling and restore the ADMA-NO pathway, to protect against HF/HS-induced programmed hypertension.

Our study has a few limitations. Firstly, postnatal insults (i.e., high-salt) can act as a “second hit” to deteriorate earlier programming induced by a first hit (i.e., maternal high-fructose). Thus, we conducted both one-hit models—HF/ND and ND/HS for the sake of comparison to the two-hit HF/HS model in the current study. We did not conduct a ND/ND+M group, as melatonin therapy has a remarkably benign safety profile [2]. However, the long-term programming effects of maternal melatonin therapy in the ND/ND, HF/ND, and ND/HS group deserve further clarification. However, whether maternal melatonin therapy might provoke long-term programming changes leading to adverse effects in adulthood remains to be clarified. Secondly, different nutritional insults might not mediate the same pathway to inducing hypertension that was protected by maternal melatonin therapy. Therefore, additional studies are required using other models to determine whether the nutrient-sensing signals and ADMA/NO pathway are common targets for preventing hypertension. The final limitation is that we did not examine other organs contributing to hypertension, such as the vasculature, heart, and brain. The beneficial effects of maternal melatonin therapy might be derived from other organs involved in BP regulation.

## 4. Materials and Methods 

### 4.1. Animal Models

All animal experiments were carried out in strict accordance with the recommendations of the Guide for the Care and Use of Laboratory Animals of the National Institutes of Health. This study was approved by the Institutional Animal Care and Use Committee of the Kaohsiung Chang Gung Memorial Hospital. Virgin Sprague-Dawley (SD) rats (12–16 weeks old) were obtained from BioLASCO Taiwan Co., Ltd. (Taipei, Taiwan) and housed in an Animal Care International (AAALAC)-approved animal facility in our hospital with controlled temperature and light cycle (12/12 light cycle). Male SD rats were kept with individual females until mating was confirmed by the examination of a vaginal plug. Pregnant SD rats received a normal diet (ND; *n* = 4) or a high-fructose diet (60% fructose; HF; *n* = 8) during the entire period of pregnancy and lactation [13,14]. In order to equally receive maternal pup care and quantity of milk, litters were standardized to eight pups per litter at birth. Only male offspring were selected from each litter and used in subsequent experiments, as males are prone to develop hypertension at a higher rate and at an earlier age compared to females [38]. Male offspring were assigned to five groups (maternal diet/post-weaning diet; *n* = 8 for each group): ND/ND, HF/ND, ND/HS, HF/HS, and HF/HS+M. Male offspring rats were administered either a normal diet with regular chow (ND) or a high-salt diet (HS; 1% NaCl in drinking water) from weaning to 3 months of age. In addition to HF/HS diets, mother rats in the HF/HS+M group received 0.01% melatonin in drinking water during the entire pregnancy and lactation (i.e., a total of 6 weeks). The dose of melatonin was adopted based on our previous study [34]. Melatonin was prepared twice weekly by dissolving the drug (10 mg) in 1 mL of 100% ethanol. This solution was then diluted with water to a final concentration of 0.01%. Water bottles were wrapped with aluminum foil to protect from light. 

BP was measured in conscious and previously trained offspring by using an indirect tail-cuff method (BP-2000, Visitech Systems, Inc., Apex, NC, USA) at 4, 6, 8, 10, and 12 weeks of age as previously described [39]. Three stable consecutive measures were taken and averaged. All rats were killed at 12 weeks of age. Rats were anesthetized using an intraperitoneal injection of ketamine (50 mg/kg) and xylazine (10 mg/kg), then euthanized by an intraperitoneal overdose of pentobarbital. Heparinized blood samples were collected. Kidneys were harvested after perfusion with PBS, divided into cortex and medulla regions, and snap-frozen until analysis.

### 4.2. High-Performance Liquid Chromatography (HPLC)

The levels of several components of the NO pathway, including l-citrulline, l-arginine, ADMA, and SDMA, were measured using HPLC with the *o*-phtalaldehyde-3-mercaptoprionic acid derivatization reagent described previously [38]. Standards contained concentrations of 1–100 mM l-citrulline, 1–100 mM l-arginine, 0.5–5 mM ADMA, and 0.5–5 mM SDMA. The recovery rate was approximately 95%.

### 4.3. Quantitative Real-Time Polymerase Chain Reaction (PCR)

RNA was extracted from the kidney cortex according to previously described methods [39]. Numerous genes related to the nutrient sensing signaling pathway were analyzed, including *Sirt1*, *Sirt4*, *Prkaa2*, *Prkab2*, *Prkag2*, *Ppara*, *Pparb*, *Pparg*, and *Ppargc1a*. Additionally, *Nrf2*, a key transcription factor that regulates antioxidant defense [21], was analyzed. The 18S rRNA gene (*Rn18s*) was used as a reference in all analyses. Primer sequences are listed in Table 3. To quantify the relative gene expression, the comparative threshold cycle (C_T_) method was employed. For each sample, the average C_T_ value was subtracted from the corresponding average *Rn18s* value, calculating the ΔC_T_. ΔΔC_T_ was calculated by subtracting the average control ΔC_T_ value from the average experimental ΔC_T_. The fold-increase of the experimental sample relative to the control was calculated using the formula 2^−ΔΔCT^.

### 4.4. Western Blot

Western blot analysis was performed using the methods published previously [37]. Briefly, samples (200 μg of kidney cortex) were loaded on a 6–10% polyacrylamide gel and separated by electrophoresis (200 volts, 90 min). Following transfer to a nitrocellulose membrane (GE Healthcare Bio-Sciences Corp., Piscataway, NJ, USA), the membranes were incubated with Ponceau S red (PonS) stain solution (Sigma-Aldrich, St. Louis, MO, USA) for 10 minutes on the rocker. After blocking with phosphate-buffered saline-Tween (PBS-T) containing 5% dry milk, the membranes were incubated with primary antibody. We used the following primary antibodies: goat polyclonal anti-rat AMPKα2 antibody (1:1000, overnight incubation; Santa Cruz Biotechnology, Santa Cruz, CA, USA), rabbit polyclonal anti-rat PGC-1α antibody (1:1000, overnight incubation; Santa Cruz Biotechnology), rabbit polyclonal anti-rat mTOR antibody (1:1000, overnight incubation; Cell Signaling, Danvers, MA, USA) and rabbit polyclonal anti-rat phosphorylated mTOR antibody (1:1000, overnight incubation; Cell Signaling). Following five washes with 0.1% Tween-Tris-buffered saline (TBS-T), the membranes were incubated for 1 h with horseradish peroxidase-labeled secondary antibody diluted 1:1000 in TBS-T. Bands were visualized using SuperSignal West Pico reagent (Pierce; Rockford, IL, USA) and quantified by densitometry as integrated optical density (IOD), normalized to PonS staining to correct for variations in total protein loading and for an internal standard. The protein abundance was represented as IOD/PonS.

### 4.5. Statistical Analysis

Statistical analysis was conducted with one-way analysis of variance (ANOVA) with a Tukey post hoc test for multiple comparisons. BP was analyzed by two-way repeated-measures ANOVA with a Tukey post hoc test. All values are reported as mean ± SEM with the *p* value was less than 0.05 considered statistically significant. All analyses were performed using the Statistical Package for the Social Sciences software (SPSS, Chicago, IL, USA). 

## 5. Conclusions

In conclusion, maternal melatonin therapy attenuated hypertension programmed by maternal high fructose consumption combined with post-weaning high salt consumption. The beneficial effects of maternal melatonin therapy protect offspring against HF/HS-induced hypertension, including increased mRNA expression of *Sirt1*, *Sirt4*, *Prkaa2*, *Prkab2*, *Pparg*, *Ppargc1a*, and *Nrf2*, increased protein levels of AMPKα2, PGC-1α, mTOR, and phosphorylated mTOR, decreased plasma ADMA and SDMA levels, and increased the l-arginine-to-ADMA ratio. Emerging evidence is showing the benefits of melatonin in the treatment of many human diseases. There is thus a strong requirement to reconcile the reprogramming effects of maternal melatonin therapy in the protection of hypertension of developmental origin, especially for pregnant women and their children exposed to excessive dietary fructose and salt consumption.

## Figures and Tables

**Table 1:** Summary of weight, blood pressures, and functional parameters in male offspring exposed to maternal high fructose intake, post-weaning high-salt diet, and melatonin at 12 weeks of age.

Groups	ND/ND	HF/ND	ND/HS	HF/HS	HF/HS+M
Mortality	0%	0%	0%	0%	0%
BW (g)	432 ± 15	432 ± 20	492 ± 8 ^a,b^	479 ± 13 ^a,b^	435 ± 15 ^c,d^
Left kidney weight (g)	1.67 ± 0.11	1.75 ± 0.08	2.14 ± 0.05 ^a,b^	2.19 ± 0.08 ^a,b^	1.81 ± 0.09 ^c,d^
Left kidney weight/100 g BW	0.39 ± 0.02	0.41 ± 0.01	0.44 ± 0.01	0.46 ± 0.01	0.42 ± 0.01
Systolic blood pressure (mm Hg)	135 ± 2	154 ± 3 ^a^	162 ± 1 ^a^	174 ± 2 ^a,b^	152 ± 1 ^b,c,d^
Diastolic blood pressure (mm Hg)	82 ± 2	80 ± 2	85 ± 2	97 ± 6 ^a,b,c^	72 ± 3 ^a,b,c,d^
Mean arterial pressure (mm Hg)	100 ± 1	105 ± 1 ^a^	111 ± 2 ^a^	122 ± 4 ^a,b,c^	99 ± 2 ^b,c,d^

HF/ND, maternal high fructose intake; ND/HS, post-weaning high salt intake; HF/HS, maternal high fructose plus post-weaning high salt intake; HF/HS+M, maternal high fructose plus post-weaning high salt intake and treated with melatonin. BW, body weight; ^a^ *p* < 0.05 vs. ND/ND; ^b^ *p* < 0.05 vs. HF/ND; ^c^ *p* < 0.05 vs. ND/HS; ^d^ *p* < 0.05 vs. HF/HS. As shown in Figure 1, maternal HF diet induced a rise in SBP from 8 to 12 weeks of age. Additionally, the SBP significantly increased in ND/HS as well as in HF/HS group compared with that in ND/ND group from week 4 through 12. The increases of SBP in the HF/HS group was reduced by maternal melatonin therapy from 4 to 12 weeks of age. These data indicated that post-weaning HS intake aggravated maternal HF diet induced programmed hypertension in adult male offspring, which melatonin prevented.

**Table 2:** Plasma levels of l-arginine, l-citrulline, ADMA, and SDMA in male offspring exposed to maternal high fructose intake, post-weaning high-salt diet, and melatonin at 12 weeks of age.

Groups	ND/ND	HF/ND	ND/HS	HF/HS	HF/HS+M
l-citrulline	57.2 ± 1.1	49.1 ± 1.6	95.2 ± 2.8 ^a,b^	85.8 ± 1.8 ^a,b^	45 ± 1.6 ^c,d^
l-arginine	288.3 ± 6.7	208 ± 5.8 ^a^	285.2 ± 3.0 ^b^	287.4 ± 5.8 ^b^	221.9 ± 2.7 ^a,c,d^
ADMA	1.01 ± 0.03	1.05 ± 0.05	2.34 ± 0.04 ^a,b^	2.3 ± 0.09 ^a,b^	1.48 ± 0.09 ^c,d^
SDMA	0.61 ± 0.01	0.59 ± 0.01	3.12 ± 0.12 ^a,b^	2.84 ± 0.08 ^a,b^	0.55 ± 0.04 ^c,d^
l-arginine-to-ADMA ratio	226.2 ± 3.0	201.9 ± 11.3	123.1 ± 2.6 ^a,b^	128.9 ± 3.8 ^a,b^	191 ± 24 ^c,d^

ADMA, asymmetric dimethylarginine; SDMA, symmetric dimethylarginine; HF/ND, maternal high fructose intake; ND/HS, post-weaning high salt intake; HF/HS, maternal high fructose intake plus post-weaning high salt intake; HF/HS+M, maternal high fructose intake plus post-weaning high salt intake and treated with melatonin; ^a^ *p* < 0.05 vs. ND/ND; ^b^ *p* < 0.05 vs. HF/ND; ^c^ *p* < 0.05 vs. ND/HS; ^d^ *p* < 0.05 vs. HF/HS.

**Table 3:** Quantitative real-time polymerase chain reaction primer sequences.

Gene	Forward	Reverse
*Sirt1*	5 tggagcaggttgcaggaatcca 3	5 tggcttcatgatggcaagtggc 3
*Sirt4*	5 ccctttggaccatgaaaaga 3	5 cggatgaaatcaatgtgctg 3
*Prkaa2*	5 agctcgcagtggcttatcat 3	5 ggggctgtctgctatgagag3
*Prkab2*	5 cagggccttatggtcaagaa 3	5 cagcgcatagagatggttca 3
*Prkag2*	5 gtgtgggagaagctctgagg 3	5 agaccacacccagaagatgc 3
*Ppara*	5 agaagttgcaggaggggatt 3	5 ttcttgatgacctgcacgag 3
*Pparrb*	5 gatcagcgtgcatgtgttct 3	5 cagcagtccgtctttgttga 3
*Pparg*	5 ctttatggagcctaagtttgagt 3	5 gttgtcttggatgtcctcg 3
*Ppargc1a*	5 cccattgagggctgtgatct 3	5 tcagtgaaatgccggagtca 3
*Nrf2*	5 cccattgagggctgtgatct 3	5 tcagtgaaatgccggagtca 3
*Rn18s*	5 gccgcggtaattccagctcca 3	5 cccgcccgctcccaagatc 3
